# A Rare Case of Severe Burns in a Pregnant Woman

**DOI:** 10.7759/cureus.76076

**Published:** 2024-12-20

**Authors:** Ana Pflaum, Lilijana Kornhauser Cerar, David Lukanovic, Uros Ahcan, Leon Meglic

**Affiliations:** 1 Department of Gynecology, Division of Gynecology and Obstetrics, Ljubljana University Medical Centre, Ljubljana, SVN; 2 Department of Perinatology, Division of Gynecology and Obstetrics, Ljubljana University Medical Centre, Ljubljana, SVN; 3 Department of Plastic Surgery and Burns, Ljubljana University Medical Centre, Ljubljana, SVN

**Keywords:** burns, case report, newborn death, pregnancy, trauma burns

## Abstract

Although burns are an extremely rare injury during pregnancy, they place a significant additional burden on the body, which is physiologically adapted to pregnancy and therefore limited in its ability to respond effectively to stress. Due to the low incidence of burns during pregnancy, the existing literature is scarce. Case reports are mostly from third-world countries, and there are no official guidelines or recommendations. This case report describes a 30-week pregnant woman who sustained superficial dermal burns covering up to 45% of the total body surface area (TBSA) and received appropriate fluid resuscitation therapy and wound management according to international guidelines. A neonate was delivered by emergency cesarean section after completion of antenatal corticosteroid therapy but died after 10 days due to hypoxic damage to the brain, kidneys, and digestive system. The patient was discharged after 51 days with no long-term sequelae. This case is unique in its outcome and should be used as an important reminder to take into account the physiological adaptations of the body to pregnancy when optimizing the management of burn injuries and to emphasize the importance of adequate fluid resuscitation, careful pregnancy monitoring, and timely decision-making about possible preterm labor with emergency cesarean sections.

## Introduction

Burn injuries, though increasingly rare in developed countries, remain a significant cause of morbidity and prolonged hospitalization [1, 2]. Among pregnant women, the incidence is 0.17 per 100,000 person-years. Physiological adaptations during pregnancy, such as increased cardiac output and altered vascular resistance, heighten maternal and fetal vulnerability, leading to higher mortality and morbidity in this population [2, 3]. Burns in pregnancy can result in adverse outcomes, including preterm delivery, stillbirth, and neonatal complications [2, 4].

Effective management of burns during pregnancy necessitates a multidisciplinary approach. General burn care principles apply, with modifications to account for teratogenic risks and physiological changes. This case report contributes valuable insights into the management of severe burns in pregnancy, emphasizing the critical role of tailored fluid resuscitation, coordinated multidisciplinary care, and timely obstetric interventions [5].

This case report presents the clinical management and outcomes of a rare and complex case of severe burn injury in a pregnant patient, emphasizing the challenges of balancing maternal and fetal needs. In the absence of formal guidelines for such cases, it offers valuable insights to bridge gaps in the existing literature. The report underscores the critical importance of a multidisciplinary approach, involving obstetrics, anesthesiology, neonatology, and plastic surgery, to optimize outcomes. It highlights the necessity of fluid resuscitation tailored to the physiological demands of pregnancy, ensuring maternal hemodynamic stability and fetal health. Timely decisions regarding preterm delivery, guided by antenatal corticosteroid administration and close fetal monitoring, were pivotal. This case reinforces the urgent need for further research to develop evidence-based guidelines for managing similar scenarios.

## Case presentation

A 36-year-old woman at 29 weeks of her second pregnancy was admitted to the intensive therapy unit (Department of Plastic, Reconstructive, Aesthetic Surgery, and Burns) of the University Clinical Centre in Ljubljana, Slovenia, following extensive burns to the lower body. The injury occurred at home when a central heating boiler exploded, throwing her to the floor.

**Figure 1:**
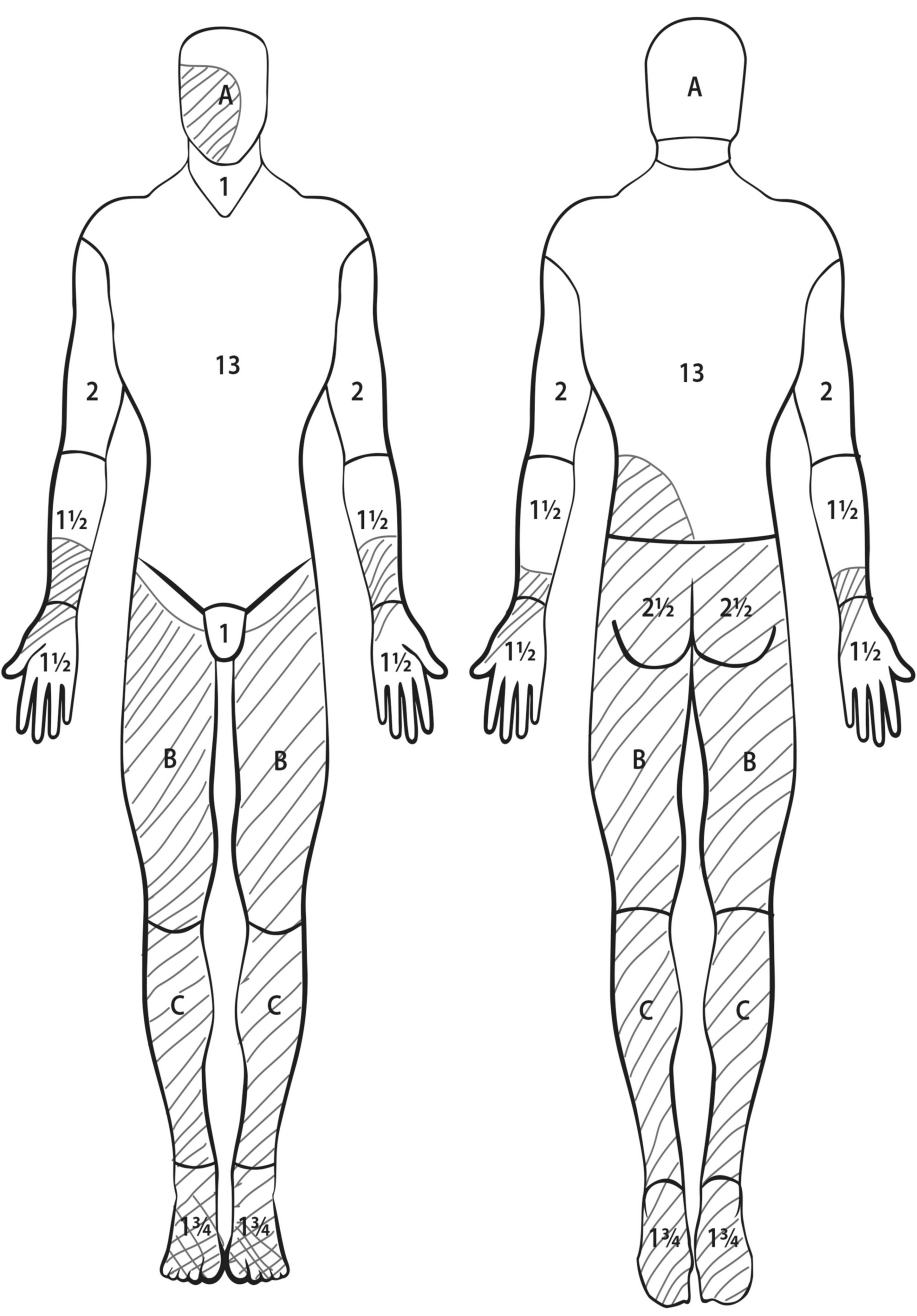
The areas of the patient's body that were burned This figure has been created by the authors.

On admission, she was fully conscious, intubated, and breathing spontaneously with a 40% oxygen mask. She was analgosedated but contactable, with vital signs showing blood pressure of 120/70 mmHg, heart rate 120/min, respiratory rate 12/min, Glasgow Coma Scale (GCS) score of 12, and body temperature of 36°C. Physical examination revealed predominantly superficial dermal burns covering 45% of the total body surface area (TBSA), affecting the lower back, gluteal regions, and lower extremities (Figure 1). No skeletal trauma was identified. Chest and pelvic radiographs, as well as abdominal ultrasound, confirmed a viable pregnancy with normal fetal growth for gestational age. The fetus was transverse in position. The patient received two doses of betamethasone (14 mg/24 hours), and a premature cesarean section was planned after completing corticosteroid therapy.

Supportive care included analgesia, anticoagulation with dalteparin (5000 IU/24 h), and fluid resuscitation following the Parkland formula for the first 48 hours, transitioning to the modified Brook formula (Table 1).

**Table 1:** Fluid resuscitation in the first 48 hours

	Day of admission	Day 1
Colloids	1300 ml	950 ml
Crystalloids	10752 ml	6117 ml
Per OS	120 ml	440 ml
Sum	9672 ml	2762 ml

On the first day, the patient reported absent fetal movements. Abnormal cardiotocography (CTG) prompted a multidisciplinary decision to perform an emergency cesarean section. The procedure was uncomplicated, with an estimated blood loss of 200-300 mL.

A baby girl was delivered. The baby's birth measurements are showcased in Table 2. Although initially crying, she required immediate intubation and oxygen support before her transfer to the neonatal intensive care unit. On admission, early signs of hydrops fetalis, including peripheral edema, pleural effusion, and ascites, were observed. Echocardiography revealed severe tricuspid regurgitation, though neonatal blood pressure and cardiac output remained normal. Treatment included surfactant therapy, furosemide for diuresis, and prophylactic antibiotics, later discontinued following negative culture results.

**Table 2:** Immediate after-birth measurements of the baby APGAR: appearance, pulse, grimace, activity, and respiration; BE: base excess

Measurement	Value
Weight	1600 g
Length	41.5 cm
Head circumference	28.5 cm
APGAR	5, 6, 7
pH (arterial/BE)	7.18 / -8.7
pH (venous/BE)	7.37 / -6.2

Cranial ultrasound (US) and MRI of the baby revealed extensive hypoxic-ischemic brain injury, including large left perimedullary venous infarction, thrombosed veins, and ventricular hemorrhage. Disseminated intravascular coagulopathy necessitated plasma and erythrocyte transfusion. Abdominal ultrasound identified a large aortic thrombus. The neonate developed multi-organ failure, including severe hypoxic brain, renal, and gastrointestinal damage, and succumbed on the tenth day of life in her parents’ arms.

Postpartum, the patient underwent daily burn wound dressings with saline and advanced wound care using Aquacel Ag, silver sulfadiazine, and dexpanthenol. Periodic surgical debridement and skin grafting were performed under general anesthesia. She received respiratory and physical rehabilitation, with psychological support to process the loss. After 51 days, she was discharged in good condition, with instructions for continued wound care and physiotherapy.

## Discussion

The available literature on burns during pregnancy is sparse, with most studies originating from low-resource settings where over 90% of burn cases occur [6]. Burns rank as the fourth most common type of injury globally, following road traffic accidents, falls, and violence. A review of PubMed identified only case reports and retrospective studies [4, 7-9], which consistently reported a low incidence of burns in pregnancy but alarmingly high maternal and fetal mortality rates when burns exceed 50% of the TBSA. Fetal mortality is highest in the first trimester, while maternal mortality peaks in the third trimester. Only one study has provided specific guidance on fluid resuscitation, pregnancy monitoring, and decision-making regarding the continuation of pregnancy or induction of labor [9].

Burn injuries are caused by thermal, chemical, or electrical energy and vary in depth, affecting hemodynamic, homeostatic, respiratory, and metabolic systems. They trigger a severe systemic inflammatory response, which can lead to sepsis, multi-organ failure, and, in extreme cases, death. Burns are classified by depth (epidermal, superficial dermal, deep dermal, and subdermal) and by the percentage of TBSA affected, with severity increasing proportionally to the depth and extent of injury [1].

Upon admission, the cause, size, and depth of the burn must be determined. Patients with burns exceeding 20% TBSA are prone to significant intravascular fluid loss, necessitating intensive fluid resuscitation. Fluid requirements are typically calculated using the Parkland or modified Brook formulas [5].

Treatment principles for pregnant burn victims are largely similar to those for non-pregnant patients, involving primary care, acute treatment, and rehabilitation. Initial care adheres to the ABCDEF framework (i.e., airway, breathing, circulation, disability, exposure, and fluid resuscitation and fetal evaluation). Acute treatment includes fluid resuscitation, analgesics, antibiotics, and surgical intervention, while rehabilitation, often overlapping with acute care, can extend over several years [3, 5].

Pregnancy introduces unique physiological changes, particularly in the uterine vascular system. By the third trimester, uterine blood flow reaches 600 mL/min and lacks autoregulation, making placental perfusion highly sensitive to maternal blood pressure fluctuations. Reduced placental perfusion can result in fetal hypoxia and acidosis. Maternal hypovolemia also decreases amniotic fluid volume, heightening the risk of fetal injury or intrauterine death [7, 10, 11]. Pregnant burn victims require significantly greater fluid volumes (30%-60% more than the Parkland formula predicts) to maintain hemodynamic stability, but over-resuscitation risks pulmonary edema. Crystalloids are preferred over colloids, as damaged endothelium allows fluids to leak into interstitial spaces, causing edema regardless of fluid type [2, 3, 5].

The decision to induce labor depends on burn severity, maternal and fetal status, and gestational age. Optimal outcomes occur at 32 weeks or later if no fetal hypoxia or injuries are present. At 24-30 weeks, decisions are individualized, weighing the risks and benefits of preterm delivery, delayed delivery, and antenatal corticosteroid administration. For burns exceeding 55% TBSA, immediate cesarean delivery without corticosteroids is recommended. When burns affect less than 55% TBSA, pregnancies can often be monitored with antenatal corticosteroids and delayed preterm delivery [2, 5]. In this case, with 45% TBSA burns and a gestational age of 30 weeks, the interdisciplinary team prioritized corticosteroid therapy and delayed delivery to optimize outcomes.

Despite these measures, the fetus in this case sustained ischemic damage to the central nervous system (CNS), kidneys, and digestive system due to antenatal hypoxia, culminating in coagulopathy. The newborn died on the tenth day of life despite prompt intervention. To the best of our knowledge, such outcomes have not been reported in prior literature. Antenatal hypoxia likely resulted from maternal hemodynamic instability caused by fluid shifts, systemic inflammatory responses, and compromised uteroplacental perfusion. Intensive fluid resuscitation presented challenges, with both under and over-resuscitation exacerbating fetal hypoxia. Additionally, drug therapy posed unique challenges, as the placental and blood-brain barriers limited medication efficacy and selection. For example, analgesics and antibiotics are needed to balance teratogenic risks with maternal treatment needs, while systemic inflammation increases CNS drug susceptibility.

Neonatal arterial or venous thrombosis, as observed in this case, is more common in preterm infants, particularly those exposed to traumatic deliveries, maternal preeclampsia, perinatal hypoxia, sepsis, or dehydration. Interestingly, the risk is higher in male neonates for reasons not yet understood. Here, a synergistic effect of antenatal hypoxia and dehydration in a low-gestational-age fetus likely contributed to the fatal outcome. Treatment options for neonatal thrombosis remain limited, as guidelines are lacking due to insufficient evidence-based research [12, 13].

This case underscores the complexity of managing severe burns in pregnancy, necessitating an interdisciplinary approach involving obstetrics, anesthesiology, neonatal, and surgical teams. Adjustments to standard burn protocols must account for pregnancy-specific physiological changes. By detailing rare complications and fetal outcomes, this report offers valuable insights for maternal trauma management, emphasizing the critical need for evidence-based guidelines to inform care in similar cases.

## Conclusions

The case presented is an exceptionally rare occurrence in clinical practice, posing significant challenges for all healthcare professionals involved. It highlights the critical need for a multidisciplinary approach to managing burn injuries during pregnancy, taking into account the unique physiological changes that accompany this condition. Effective care requires highly tailored strategies that balance the needs of both the mother and fetus. These include precise fluid resuscitation adapted to pregnancy-specific hemodynamic demands, meticulous monitoring of maternal and fetal well-being, and timely, evidence-informed decisions regarding the management of preterm labor and the necessity of emergency cesarean sections. This case emphasizes the importance of collaborative, interdisciplinary efforts to optimize outcomes in such complex scenarios.
